# Co-morbidity and health care utilisation five years prior to diagnosis for depression. A register-based study in a Swedish population

**DOI:** 10.1186/1471-2458-11-552

**Published:** 2011-07-12

**Authors:** David Andersson, Henrik Magnusson, John Carstensen, Lars Borgquist

**Affiliations:** 1 Department of Medical and Health Sciences, Family Medicine, Linköping University, SE 58183 Linköping, Sweden; 2 Department of Medical and Health Sciences, Health and Society, Linköping University, Linköping, Sweden

## Abstract

**Background:**

Depressive disorders have been associated with a number of co-morbidities, and we hypothesized that patients with a depression diagnosis would be heavy users of health care services, not only when first evaluated for depression, but also for preceding years. The aim of this study was to investigate whether increased health care utilisation and co-morbidity could be seen during five years prior to an initial diagnosis of depression.

**Methods:**

We used a longitudinal register-based study design. The setting comprised the general population in the county of Östergötland, south-east Sweden. All 2470 patients who were 20 years or older in 2006 and who received a new diagnosis of depression (F32 according to ICD-10) in 2006, were selected and followed back to the year 2001, five years before their depression diagnosis. A control group was randomly selected among those who were aged 20 years or over in 2006 and who had received no depression diagnosis during the period 2001-2006.

**Results:**

Predictors of a depression diagnosis were a high number of physician visits, female gender, age below 60, age above 80 and a low socioeconomic status.

Patients who received a diagnosis of depression used twice the amount of health care (e.g. physician visits and hospital days) during the five year period prior to diagnosis compared to the control group. A particularly strong increase in health care utilisation was seen the last year before diagnosis. These findings were supported with a high level of co-morbidity as for example musculoskeletal disorders during the whole five-year period for patients with a depression diagnosis.

**Conclusions:**

Predictors of a depression diagnosis were a high number of physician visits, female gender, age below 60, age above 80 and a low socioeconomic status. To find early signs of depression in the clinical setting and to use a preventive strategy to handle these patients is important.

## Background

Depression is one of the most prevalent mental disorders in the general population [1,2] and is a leading cause of disease burden in the world [3]. People with a depressive diagnosis represent a large share of patients using the health care system, especially in primary care, and their total health care utilisation is substantial. Other disorders such as musculoskeletal disorders may co-occur and precede a depressive diagnosis [4-6]. Co-morbidity is frequent in a depressive patient group [7].

Depression has also been associated with health problems such as anxiety, alcohol and drug abuse. In addition, alcohol disorders have also been suggested as an important risk factor for mood disorders. Psychosocial disorders have been pointed out as putative risk factors for depressive disorders [8]. Further, anxiety disorders and depressive disorders have been found to share a similar genetic background [9].

The cumulative life risk for a depression was 23% for men and 31% for women in the longitudinal Swedish Lundby study during the period 1972-1997 [10]. Female gender has been reported as a risk factor for depressive disorders [11]. Risk factors for depression may also be poor socioeconomic conditions. Participants with lower socioeconomic status had nearly a twofold increase in risk for major depression [12].

The use of antidepressant drugs is frequent in a general population and underreporting depressive disorders and other psychiatric disorders is common. Since the introduction of selective serotonin reuptake inhibitors (SSRI) the sales of antidepressant drugs have increased heavily. A number of studies have demonstrated the efficacy of antidepressants and psychotherapy in treatment of depression. However, there is a paucity of population-based longitudinal studies examining the relationships between depressive disorders, co-morbidity, and health care utilisation.

We hypothesized that patients who have received a depressive diagnosis would be heavy users of health care services, not only when first evaluated for a depression, but also for several preceding years.

The aim of this study was to investigate whether increased health care utilisation and co-morbidity could be seen during five years prior to an initial diagnosis of depression. Age, gender and socioeconomic status were included in the analyses as adjusting factors as well as predictors.

## Methods

### Depression group and control group

In this population-based study we used different linked population registers of the 440 000 (2006) residents of the county of Östergötland, south-east Sweden. Individual data on clinical diagnosis, socioeconomic status, antidepressant drug use, patient costs and health care utilisation were linked for the whole population. The personal identification numbers in Sweden facilitate linking information from different registers.

All 2470 patients who 1) were 20 years of age or older in 2006, 2) had received a diagnosis of depression (F32) according to the International Classification of Diseases, 10th version (ICD-10) in 2006, and 3) had not been recorded with related diagnoses (F31-F39) during the preceding five years (2001-2005) were selected. From the population we randomly selected a control group of 28500 (out of 251218) persons who were 20 years or older in 2006 and who had no diagnosis of depression during the period 2001-2006. Patients and controls were residents of the county council of Östergötland during the whole of the study period. Confidentiality was ensured by encrypted numbers and the study was approved by the Regional Ethical Review Board in Linköping.

### Health care utilisation and patient costs

#### Health care utilisation

The Care Data Warehouse in Östergötland, (CDWÖ) consists of administrative records of all publicly produced health care utilisation in the county including inpatient and outpatient care for all medical specialities (more than 95% of the health care utilisation in the county).

For all the visits to a physician and all hospitalisations in the CDWÖ, it was possible to record up to ten diagnoses for hospital care and for primary care. All health care utilisation per patient for the period 2001-2006 was extracted and expressed by the following variables: total number of visits to out-patient care including physician visits (hospital out-patient visits, all GP visits) and visits to paramedical staff. Besides, the number of hospitalisation days, in-patient care, was extracted.

#### Costs per patient (CPP)

The CPP database was linked to CDWÖ and included costs for each health care contact for every patient who had been in contact with health services. Costs have been calculated for all healthcare services, e.g. a visit to a physician, a nurse or a laboratory test. Thus, it was possible, for example, to summarize the CPP for healthcare in different clinics and for each person, over a certain period of time. We used cost data from 2005-2007 in this study. Typical unit costs were GP visits (SEK 1655), and physician psychiatric out-patient visits (SEK 2530). We also added drug costs from the Swedish prescribed Drug Register. All costs were in SEK (2007 prices).

### Drug prescriptions

The Swedish prescribed Drug Register contains all dispensed drug prescriptions and covers the whole Swedish population. All drugs are classified according to the Anatomical Therapeutic Chemical (ATC) classification system. Measurement units of utilisation are prescriptions, Defined Daily Doses (DDDs) and expenditure. The register contains data on drugs (prescribed and dispensed amount per item for each patient and costs per patient). In this study all antidepressants (N06A, according to the ATC system) were selected for the period July 2005 - December 2007 and prescribed for residents in the county of Östergötland.

### Measure of co-morbidity

Various approaches have been taken to characterize the combined burden of diseases as single measure on a scale [13]. We have used The Johns Hopkins Adjusted Clinical Groups (ACGs) Case-Mix System7.1 to express co-morbidity on an individual level [14]. This system has prior been used in Sweden [15-17] and found to have a good validity in predicting health care utilisation. The assumption is that the pattern of diagnoses, rather than single diagnoses, shows the level of co-morbidity. The aim is to take not only the presence but also the severity of different diagnoses into account. The calculations are made for diagnosis over a specified period of time, yearly in this study. All primary care and secondary care diagnoses that the patients had received during the period 2001-2006 were used in the analysis of co-morbidity level using the ACGs. Each of the individuals in the population was assigned to one of 92 ACGs and was expressed in Reference Rescaled Concurrent Weight (RRCW) according to the Case-Mix System7.1. The RRCW is constructed so that 1 is the mean of the population. A value larger than 1 means that a person is expected to cost more than average and has a higher degree of co-morbidity, while a value less than 1 indicates the opposite. A normal population (in our study the same as the control group) is expected to have a value of 1.

### Socioeconomic status

Statistics Sweden has created a Total Population Register. This register is mainly used as a basic register for preparation of statistics in the Swedish counties and municipalities regarding the size and composition of the population, stratified according to sex, age, marital status, etc. We have used individual data from this register for the variables age, gender and income.

A socioeconomic index for each resident has been created and used in the county of Östergötland, based on income for small geographical areas in the county. The index consists of five socioeconomic categories. The variable has been validated and has shown a high association with other socioeconomic variables: (the proportion of) senior citizens, immigrants, unemployed, low education, low income, block of flats and social assistance in small geographical areas [18].

### Statistical analysis

**Figure 1:**
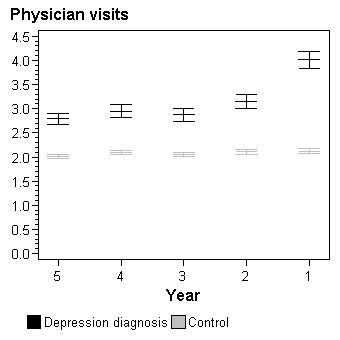
**Total number of physician visits per person by year prior to depression diagnosis**. Adjusted for gender, age and socioeconomic status.

**Figure 2:**
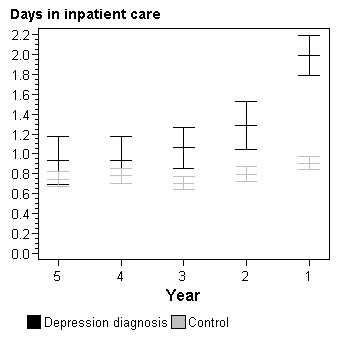
**Total number of days in inpatient care per person by year prior to depression diagnosis**. Adjusted for gender, age and socioeconomic status.

**Figure 3:**
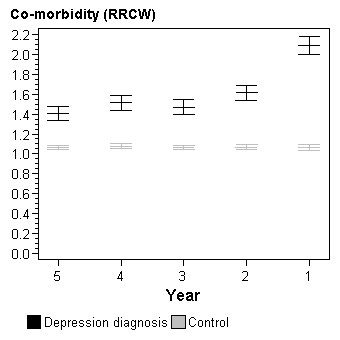
**Level of co-morbidity (RRCW) by year prior to depression diagnosis**. Adjusted for gender, age and socioeconomic status.

Pearson's Chi-Square test was used to analyse differences in the distribution of the basic variables for the group with a depression diagnosis and the control group. The mean values and confidence intervals for physician visits and co-morbidity (RRCW) in the depression and control group (Figures 1, 2, 3) were adjusted for background variables (gender, age and socioeconomic status). This was done using analysis of covariance and the means were adjusted to the distribution of background variables in the depression diagnosis group [19]. Logistic regression was used to calculate the odds ratios for a depression diagnosis (i.e. coding the response variable 1 for depression diagnosis and 0 for controls) comparing the impact of gender, age, socioeconomic status, co-morbidity (RRCW) and physician visits. We tested for all two-way interactions in order to validate the regression model.

## Results

Women were more likely to receive a depression diagnosis than men (Table 1). Patients with a depression diagnosis were both younger and older than patients in the control group but with a lower proportion of middle aged. A difference was also seen in socioeconomic status, with a higher proportion of patients with a depression diagnosis for the two lowest socioeconomic categories. Physician visits five years as well as one year prior to diagnosis were more frequent in the group with a depression diagnosis than in the controls.

**Table 1:** Background characteristics of the patients with a depression diagnosis and the control group

	Depression diagnosis (n = 2470)	Control group (n = 28500)		
	%	%	Pearson Chi-Square	P
Gender			212.9	<.0001
Women	64.1	48.8		
Men	35.9	51.2		
Age			71.2	<.0001
20-29	14.2	12.2		
30-39	17.2	16.0		
40-49	17.1	18.2		
50-59	17.0	18.5		
60-69	12.5	16.8		
70-79	10.5	10.2		
80+	11.5	8.1		
Socioeconomic status			65.2	<.0001
Highest	6.0	7.6		
Second highest	18.3	21.8		
Middle	36.5	38.9		
Second lowest	25.7	21.6		
Lowest	13.5	10.1		
Physician visits in 2001			299.6	<.0001
0	26.1	38.8		
1-2	33.8	35.5		
3-4	19.6	14.0		
5-7	12.8	7.8		
8+	7.7	3.8		
Physician visits in 2005			1162.7	<.0001
0	15.7	45.0		
1-2	29.6	27.5		
3-4	21.1	14.0		
5-7	19.8	8.5		
8+	13.9	5.0		
TOTAL	100.0	100.0		

Compared to controls and adjusted for age, gender and socioeconomic status, patients with a depression diagnosis had a higher number of physician visits per person all five years prior to the diagnosis (Figure 1). The difference was largest the last year prior to the depression diagnosis. When analysing visits to general practitioners separately from visits to specialists the results paralleled those of total physician visits (data not shown). Similar differences between the group with a depression diagnosis and the control group could be seen for the number of hospitalization days per patient (Figure 2).

The group with a depression diagnosis showed a significantly higher co-morbidity level during the whole five-year period (Figure 3). The control group was situated at RRCW level 1, which was the expected level for a normal population. The most dominant co-morbidity diagnoses emanated from the musculoskeletal system. Sixty percent of the patients with a depression diagnosis were suffering from musculoskeletal disorders during the period 2001-2006. The corresponding number for the control group was 45%. A high frequency of co-morbidity was also seen for cardiovascular diseases; 36% in the group with a depression diagnosis and 29% in the control group during the period 2001-2005. A high increase in co-morbidity was registered in the year prior to diagnosis of depression.

In multiple logistic regression analyses the likelihood of receiving a depression diagnosis in 2006 was associated with a high number of physician visits five years prior to diagnosis, low socioeconomic status, age below 60, age above 80 and female gender (Model 1, Table 2). The odds ratios for a depression diagnosis were substantially higher when the physician visits the year before diagnosis was considered (Model 2, Table 2). The physician visits five years before diagnosis was statistically significant even when adjusting for the number of visits the year before diagnosis but the odds ratios were rather low (Model 3, Table 2).

**Table 2:** Adjusted odds ratios for a depression diagnosis in relation to socio-demographic variables and prior physician visits

		Model 1		Model 2		Model 3	
		OR (95% CI)	P	OR (95% CI)	P	OR (95% CI)	P
**Gender**							
	**Men**	REF		REF		REF	
	**Women**	1.72 (1.57 - 1.87)	<.0001	1.52 (1.40 - 1.67)	<.0001	1.50 (1.37 - 1.64)	<.0001
							
**Age**			<.0001		<.0001		<.0001
	**20-29**	1.65 (1.41 - 1.94)	<.0001	1.95 (1.66 - 2.30)	<.0001	1.99 (1.68 - 2.34)	<.0001
	**30-39**	1.57 (1.34 - 1.83)	<.0001	1.82 (1.56 - 2.13)	<.0001	1.85 (1.58 - 2.16)	<.0001
	**40-49**	1.36 (1.17 - 1.59)	<.0001	1.65 (1.41 - 1.93)	<.0001	1.66 (1.42 - 1.94)	<.0001
	**50-59**	1.32 (1.13 - 1.54)	0.0004	1.44 (1.23 - 1.68)	<.0001	1.45 (1.24 - 1.70)	<.0001
	**60-69**	REF		REF		REF	
	**70-79**	1.22 (1.02 - 1.45)	0.0275	1.07 (0.90 - 1.27)	0.4683	1.06 (0.89 - 1.26)	0.5096
	**80+**	1.48 (1.25 - 1.76)	<.0001	1.31 (1.10 - 1.56)	0.0022	1.28 (1.08 - 1.52)	0.0053
							
**Socioeconomic status**			<.0001		<.0001		<.0001
	**Highest**	REF		REF		REF	
	**Second highest**	1.04 (0.86 - 1.26)	0.6905	1.04 (0.86 - 1.27)	0.6844	1.04 (0.85 - 1.26)	0.7170
	**Middle**	1.14 (0.95 - 1.36)	0.1648	1.14 (0.94 - 1.36)	0.1788	1.13 (0.94 - 1.35)	0.2052
	**Second lowest**	1.41 (1.17 - 1.70)	0.0004	1.40 (1.16 - 1.70)	0.0005	1.39 (1.15 - 1.68)	0.0007
	**Lowest**	1.53 (1.25 - 1.88)	<.0001	1.45 (1.18 - 1.78)	0.0005	1.43 (1.16 - 1.76)	0.0008
							
**Physician visits in 2001**			<.0001				0.0007
	**0**	REF				REF	
	**1-2**	1.35 (1.22 - 1.51)	<.0001			1.06 (0.95 - 1.18)	0.3074
	**3-4**	1.92 (1.69 - 2.17)	<.0001			1.26 (1.10 - 1.43)	0.0007
	**5-7**	2.25 (1.95 - 2.60)	<.0001			1.28 (1.10 - 1.49)	0.0014
	**8+**	2.63 (2.20 - 3.15)	<.0001			1.26 (1.04 - 1.52)	0.0163
							
**Physician visits in 2005**					<.0001		<.0001
	**0**			REF		REF	
	**1-2**			3.07 (2.70 - 3.49)	<.0001	2.98 (2.61 - 3.39)	<.0001
	**3-4**			4.35 (3.79 - 5.00)	<.0001	4.12 (3.57 - 4.75)	<.0001
	**5-7**			6.81 (5.89 - 7.87)	<.0001	6.34 (5.46 - 7.36)	<.0001
	**8+**			8.31 (7.07 - 9.76)	<.0001	7.57 (6.40 - 8.96)	<.0001

In further analyses all two-way interactions between ages, gender, socioeconomic status and physician visits were included in the model but none was found statistically significant. We also analysed each of the other variables related to health care utilisation (RRCW level, hospital days and total number of visits) in logistic regression models. However, the results were similar to that for physician visits (data not shown). In additional logistic regression analyses, RRCW was not significantly associated with depression diagnosis when adjusting for physician visits. It should be noted, that RRCW and physician visits were highly correlated (Spearman rho > 0.88 for each year 2001 to 2005).

The group with a depression diagnosis in our study spent almost twice the amount of health care costs the year before diagnosis compared with the control group (SEK

25600 per patient compared with SEK 13 400 per patient). The year after diagnosis the costs were more than three times higher: SEK 44300 for the patients with a depression diagnosis compared with SEK 14100 for the controls. The amount of health care costs consisted of in-patient care, out-patient care and drugs.

## Discussion

Predictors of a depression diagnosis were a high number of physician visits, female gender, age below 60, age above 80 and a low socioeconomic status.

Patients who received a diagnosis of depression used twice the amount of health care (e.g. physician visits and hospital days) during the five year period prior to diagnosis compared to the control group. A particularly strong increase in health care utilisation was seen the last year before diagnosis. These findings were supported with a high level of co-morbidity in terms of both RRCW and particular diagnoses, as for example musculoskeletal disorders, during the whole five-year period for patients with a depression diagnosis.

Co-morbidity was an important factor in order to explain the high level of resource use for patients with a depression disease. From many studies it is known that the musculoskeletal system is overrepresented in patients who later receive a depression diagnosis [4]. The same is valid for the patients with problems from the cardio-vascular system [7]. This fact was also confirmed in our study where 60% of the patients with a depression diagnosis also suffered from musculoskeletal disorders and 36% had a cardiovascular disease before getting the depression diagnosis.

Depression*s *are more common in midlife than in the elderly. However, depressive symptoms are more frequent among the oldest old explained by factors associated by aging such as co-morbidity [20]. This is also seen in our data: odds ratios were highest in young ages and decreasing in the elderly and finally with a peak in the oldest old (but with lower odds ratios than in midlife). More adjustment for co-morbidity (i.e. physician visits) reduced the depression odds ratio in the older groups illustrating that co-morbidity is an important factor especially in the oldest old (Model 3 versus Model 1, Table 2).

Psychiatric diagnoses have been found to be underreported in registers, especially in primary care [21]. Drug use, might be another important indicator of high health care utilisation and also a predictor of a future depression diagnosis. Hence, the use of drugs with a somatic indication has been found to be on a high level several years before treatment with antidepressants and prior to a psychiatric diagnosis [22].

In the study by Bingefors et al [22], only 45% of antidepressant-treated patients had a recorded psychiatric diagnosis. But when the medical records for these patients were further scrutinised 80% had depressive symptoms or a diagnosis of a depressive disorder. In our study it was shown that patients treated with antidepressants used more than twice as many health care resources as controls. Earlier studies of depression patients have shown a high concurrent use of health care resources, also for somatic complaints [23].

In our study almost 30% of the patients in the group with a depression diagnosis were treated with antidepressants during the year before diagnosis, with a majority of the patients between 45 and 60 years of age. The corresponding share of the control group was only 6%. Our finding that not all of the patients with a depression diagnosis receive antidepressants is in line with other studies [24].

Treatment with antidepressants increased to more than 80% of the patients the year after the depression diagnosis was given, compared with 7% in the control group.

This implies that 20% of the patients with a depression diagnosis were still not treated with an antidepressant drug. Some of these patients may have been treated with other alternatives such as physiotherapy or cognitive behavioural therapy.

The discrepancy between psychiatric diagnoses and the antidepressant drug use might be explained by doctors and patients behaviour. Health care providers may record the same condition with a variety of cods and it might be possible that a depressive health status was present before the depression diagnosis put in the medical records. A somatic diagnosis might have been used instead because psychiatric conditions might still be stigmatized by society and patients would perhaps rather see their primary physicians than psychiatric specialists.

There is a lack of population-based longitudinal studies examining the relationships co-morbidity, and health care utilisation. However, in a similar investigation compared to our study it was found that obstructive sleep apnea syndrome patients have symptoms, high health care utilisation and co-morbidities as far back as ten years before recognition of their disorder [25].

Strengths of this study were the use of different register databases and the linkage to other registers. The use of a personal identification number made it possible to follow individuals and there was total population coverage. The possibility to follow health care utilisation on an individual level during a long period of time even in primary care was a great advantage in this study. When using registers, sources of bias such as recall bias, response bias and selection bias could be kept at a minimum. An additional strength was the size of the study, with more than 2400 patients having a new diagnosis of depression. The study size results in a high power for the analysis of interactions and, since none of those were even close to significant, our results indicate that the stability of the associations between health care utilisation and later depression diagnosis over age groups, gender, and socio-economic status is high.

A weakness of using registers is the quality of data and the broad clinical variation, for instance variation in the definition of depression. This might create a bias and an uncertainty in the cohort of depression diagnosis.

The co-morbidity concept is complex and there is a lack of consensus about how to define and measure the concept. Other concepts are multi-morbidity, burden of disease and frailty, especially in the geriatric field [13]. Often these concepts are multidimensional and more comprehensive compared to the measure RRCW we have used in the present study. However, the ACG instrument has been used and been found of a good validity in a Swedish setting [15] and it was easy to handle in our study. In this study the number of physician visits and RRCW were highly correlated, which is not so unexpected since RRCW is constructed to predict health care utilisation. One consequence was that physician visits and RRCW could not be included in a regression model simultaneously without a considerable loss in statistical power.

There are several factors associated with the heavy health care utilisation among patients with a depression diagnosis. We adjusted for well-known factors associated with high health care utilisation such as a low socioeconomic status and female gender [26]. Female gender usually has a high use of health care resources and it is well-known that women have a high incidence and prevalence of depressive disorders [10]. A weakness of study is that we only analyzed patient-related variables and not provider-related variables such as location of health care units (rural or urban), private or public care, remuneration system, etc. which might influence health care utilisation. However the Swedish health care system and the County Council of Östergötland was (in 2006) rather homogenous, geographically and economically, and was dominated by public providers and a health care personnel who was salaried.

Patients with a depressive diagnosis represent a large share of patients in health care, especially in primary care. GPs are nowadays confronted with an increasing multi morbidity of an aging population and they are under pressure of psychiatric hospitals which seek to integrate their patients in the local community. The organizational format is to attach different professionals to GP practices. A central obstacle in Swedish health care is that clinical services are the responsibility of county council while care for social needs is the responsibility of the local municipalities. Besides, there is no GP gate-keeper system in Sweden. However, a new reform: "choice of care" for citizens has been introduced within primary care where different actors, both public and private, provide services as long as certain quality criteria are met. Today primary care services are therefore provided by a mix of public and private actors which may influence clinical management and sometimes create delays in the health care processes. However, there are also recent organisational incentives to convince primary care, specialist care and social care to create "chains of care" for chronically ill persons and patients with multi morbidity.

In this study we found that early signs of a depression diagnoses were a high number of physician visits, female gender, age below 60, age above 80 and a low socioeconomic status. The incentives to create chains of care might therefore include and integrate our findings in order to prevent delays in the clinical patient management. Early diagnosis and treatment of patient with depressive disorders would improve the health situation of these patients and may reduce the financial burden of health care providers and taxpayers. The effectiveness of pharmacological treatment of depressive disorders is generally accepted, and most patients who are treated experience a strong improvement in their quality of life and are able to take part in a daily working life.

To conclude, the high level of co-morbidity during the whole five-year period for patients with a new depression diagnosis had a heavy impact on health care utilisation. Caution should be exercised in the GP surgery when meeting a young or very old woman with a low socioeconomic status and many physician visits within a short time-period. Early detection and adequate treatment of these patients may reduce high health care utilisation and improve the quality of life for this group of patients.

## Conclusions

Predictors of a depression diagnosis were a high number of physician visits, female gender, age below 60, age above 80 and a low socioeconomic status. To find early signs of depression in the clinical setting and to use a preventive strategy to handle these patients is important.

## Competing interests

The authors declare that they have no competing interests.

## Authors' contributions

DA compiled data files, made the statistical analyses, drafted the manuscript and prepared the final version of the manuscript. HM compiled the data files, analysed the data, revised the manuscript. JC analysed the data, revised the manuscript critically for important intellectual content. LB initiated the study, wrote major parts of the manuscript, revised the manuscript critically. All authors read and approved the final manuscript.

## Pre-publication history

The pre-publication history for this paper can be accessed here:

http://www.biomedcentral.com/1471-2458/11/552/prepub
